# Molecular Identification and Genetic Characterization of *Macrophomina phaseolina* Strains Causing Pathogenicity on Sunflower and Chickpea

**DOI:** 10.3389/fmicb.2017.01309

**Published:** 2017-07-19

**Authors:** Ali N. Khan, Faluk Shair, Kamran Malik, Zafar Hayat, Muhammad Ayub Khan, Fauzia Yusuf Hafeez, Muhammad Nadeem Hassan

**Affiliations:** ^1^COMSATS Institute of Information Technology Islamabad, Pakistan; ^2^Oilseed Section, National Agriculture Research Council Islamabad, Pakistan

**Keywords:** *Macrophomina phaseolina*, diversity, pathogenicity, RAPD

## Abstract

*Macrophomina phaseolina* is the most devastating pathogen which causes charcoal rot and root rot diseases in various economically important crops. Three strains *M. phaseolina* 1156, *M. phaseolina* 1160, and *M. phaseolina* PCMC/F1 were tested for their virulence on sunflower (*Helianthus annuus* L.) and chickpea (*Cicer arietinum* L.). The strains showed high virulence on both hosts with a disease score of 2 on chickpea and sunflower. The strains also increased the hydrogen per oxide (H_2_O_2_) content by 1.4- to 1.6-fold in root as well as shoot of chickpea and sunflower. A significant increase in antioxidant enzymes was observed in fungal infected plants which indicated prevalence of oxidative stress during pathogen propagation. The *M. phaseolina* strains also produced hydrolytic enzymes such as lipase, amylase, and protease with solubilization zone of 5–43 mm, 5–45 mm, and 12–35 mm, respectively. The *M. phaseolina* strains were identified by 18S rRNA and analyzed for genetic diversity by using random amplified polymorphic DNA (RAPD) markers. The findings based on RAPD markers and 18S rRNA sequence analysis clearly indicate genetic variation among the strains collected from different hosts. The genetically diverse strains were found to be pathogenic to sunflower and chickpea.

## Introduction

*Macrophomina phaseolina* is a soil borne phytopathogenic fungus having a wide host range of about 500 cultivated and wild plant species worldwide (Khan, 2007). Important diseases caused by *M. phaseolina* include color rot, damping off, charcoal rot, stem rot, root rot, and seedling blight in economically important crops (Babu et al., 2007). The plants affected with fungus show necrotic lesion on different parts such as branches, peduncles, and stems. A higher temperature and low moisture favors the disease development (Aegerter et al., 2000). The microsclerotia of the pathogen can survive on infected plant debris and soil for a long period, i.e., 2–15 years depending on the environmental conditions (Baird et al., 2003; Vasebi et al., 2013). Microsclerotia are usually spherical, black, and oblong. However, there is a great variation in their shape and size depending on substrate, isolates, and temperature (Khan, 2007).

*Macrophomina phaseolina* affects the plant by secreting an array of cell wall degrading enzymes which depolymerize the cell wall components such as cellulose, xylan, pectin, polygalacturonic acid, and other proteins (Javaid and Saddique, 2012). The most significant enzymes secreted by *M. phaseolina* are pectinases, xylanases, cellulases, and proteases (Tonukari, 2003). Pectinases break the pectin of the host cell and use carbon as a source of pathogenesis whereas exo- and endopolygalacturonase are produced in early pathogenesis and colonization of host tissues (Murad and Azzaz, 2011). Lipases are also produced by *M. phaseolina* and cause hydrolysis of the fats, mono and diglycerides into free fatty acids and glycerol (Kakde and Chavan, 2011). It also produces certain toxins such as phaseolinone and botryodiplodin which facilitate the infection (Ramezani, 2008; Bressano et al., 2010). The production of hydrolytic enzymes have been reported to play a crucial role in the development of disease (Kaur et al., 2012).

Any pathogen’s attack stimulates the plant defense mechanism through hypersensitive response (HR). As a result of HR, reactive oxygen species (ROS) such as hydroxyl radicals, superoxide radicals, and hydrogen peroxide (H_2_O_2_) are produced. The unbalanced production of ROS could damage the plants severely. Hence, the production of ROS stimulates the deployment of antioxidant enzymes which scavenge ROS to maintain a balance. Thus, activity of antioxidant enzymes could serve as good marker for estimating oxidative stress in certain plant caused by pathogen (Anthony et al., 2017).

Morphological identification of *M. phaseolina* is very difficult and often not reliable because of the variation among isolates (Babu et al., 2010b; Saleh et al., 2010). Biochemical and serological techniques are being employed to identify the fungus but their specificity is limited (Srivastava and Arora, 1997). Advances in molecular techniques have provided alternative techniques for the reliable identification of fungi. Internal transcribed spacers (ITS) and 18S rRNA have been one of the most conserved genes used to identify the fungus (Babu et al., 2010b).

Genetic techniques such as random amplified polymorphic DNA (RAPD), restriction fragment length polymorphism (RFLP), and amplified fragment length polymorphism (AFLP) have helped researchers to understand more about the genetic variation in *M. phaseolina* (Mayék-Pérez et al., 2001; Su et al., 2001; Purkayastha et al., 2006; Reyes-Franco et al., 2006). Variation in pathogenicity, physiology, morphology, and genotype of *M. phaseolina* have been reported widely (Jana et al., 2003; Edraki and Banihashemi, 2010). Genetic and pathogenic variation in *M. phaseolina* strains have been assessed in numerous isolates but so far, it is hard to discriminate of *M. phaseolina* isolates from exact hosts or geographic locations due to heterogeneous nature. Lack of a strong association between the geographical origin and genotype propose high diversity among *M. phaseolina* strains (Jana et al., 2005).

The housekeeping gene 18S rRNA sequence is an ideal marker to identify the fungi at genus level but has some limitations to discriminate the intra species of fungi. The sequence of hyper-variable regions like V2, V4, V7, and V9 play an important role in identification of fungi. The region V7 has been utilized to discriminate various strains by phylogenetic analysis (Wu et al., 2015).

Random amplified polymorphic DNA are useful markers to measure genetic relatedness and variation within and among various fungi thus facilitating the understanding of their ecology (Kini et al., 2002; Cramer et al., 2003). The present study aims to identify the virulent strains of *M. phaseolina* showing cross host pathogenicity and characterize their genetic diversity by inferring their phylogenetic lineage and RAPD markers.

## Materials and Methods

### Strain and Culture Conditions

Three strains of *M. phaseolina* strain 1156, 1161, and PCMC-F1 isolated from sesame, cowpea, and cotton were obtained from first fungal bank, University of Punjab, Lahore, Pakistan. The strains were routinely grown on potato dextrose agar (PDA) (oxoid) at 28 ± 2°C and preserved in slants containing 2% PDA.

### Detection of Hydrolytic Enzymes

Production of various hydrolytic enzymes such as protease, amylase, and lipase was detected on specific medium as described by Schinke and Germani (2012). For protease activity, the fungal strains were grown on skim milk agar. Amylase activity was detected on Pontecorvo’s minimal medium amended with 1% w/v soluble starch, respectively. Solubilization zone of amylase was detected by spreading the 1% lugol solution on the plates, respectively. Productions of lipases were detected with sterilized rhodamine B agar amended with 1% (v/v) filter sterilized olive oil. The inoculated plates were incubated at 28 ± 2°C for 4–5 days. The production zones of protease on gelatin and amylase on soluble starch were detected by pouring saturated solution of ammonium sulfate and 1% lugol solution, respectively. The zone of lipase production was observed on the basis of yellow–orange color appeared under UV light while that of protease on skim milk agar was observed as transparent area appeared after casein degradation. The experiments were repeated thrice.

### Pathogenicity of *M. phaseolina* Strains under Net House (Pot Experiment)

Pathogenicity of three strains *M. phaseolina* 1156, *M. phaseolina* 1161, and *M. phaseolina* PCMC-F1 was tested on its two hosts, i.e., chick pea (*Cicer arietinum*) and sunflower (*Helianthus annuus* L.) under net house conditions.

#### Chick Pea (*Cicer arietinum*)

Pathogenicity test was conducted on the chickpea variety Sheenghar. Seeds were obtained from Agriculture Research Station Ahmad Wala, Karak, Khyber Pakhtunkhwa Khah (KPK), Pakistan and sown in pots in net house conditions. The experiment was laid out as complete randomized design (CRD). There were three replication per treatment and four plants per replication. A 9 mm disk of each strain of *M. phaseolina* was grown on Richard’s liquid medium at 28 ± 2°C for 14 days. The fungus was drenched near plant roots by using disposable syringes at 30th day of sowing. The disease was scored by using the scale (0–2) at 40th day post inoculation by measuring the damaged root (Bhattacharyya et al., 1985). Growth retardation was observed by measuring different growth parameters such as shoot (SL) and root lengths (RL), shoot dry weight (SDW) and root dry weight (RDW).

#### Sunflower (*Helianthus annuus* L.)

Sunflower seeds of Variety SMH0917 were obtained from National Agriculture Research Centre (NARC), Islamabad, Pakistan and sown in pots under net house conditions. The experiment was laid out as described in Section “Chick Pea (*Cicer arietinum*).” The *M. phaseolina* strains were inoculated as described in Section “Pathogenicity of *M. phaseolina* Strains under Field (Field Experiment).” However, a syringe was used instead of toothpick. The growth retardation of sunflower plants infected with *M. phaseolina* strains was observed by measuring root length (RL), shoot length (SL), fresh weight and dry weight of shoots and roots. The disease was scored (0–2) on the basis of root and shoot damage.

### *In Planta* Pathogenicity Mechanism

*In planta* pathogenicity mechanism of *Macrophomina phaseolina* was explored by quantifying the ROS and their scavengers. Oxidative stress related metabolites such as H_2_O_2_ and ROS scavenging enzymes like peroxidase (POD), ascorbate peroxidase (APX), and catalase (CAT) were quantified from the roots and shoots of chickpea and sunflower. Root and shoot of 5- to 6-week-old plants were sampled and weighed. A 0.5 g of each part was grinded to fine powder in mortar pestle with liquid nitrogen. The grinded powder was dissolved in 10 mL of 100 mM phosphate buffer (pH 7.0) and centrifuged at 10,000 rpm for 20 min. The supernatant was used as crude extract.

#### Hydrogen Peroxide (H_2_O_2_)

Amount of H_2_O_2_ was quantitatively estimated as described by Jana and Choudhuri (1981). A 3 mL of crude extract was mixed with 1 mL of solution containing titanium sulfate (0.1%) and sulfuric acid (20%). The mixture was centrifuged at 10,000 rpm for 15 min. A yellow color was developed in solution whose intensity was measured at 410 nm (Singh and Jha, 2016).

#### Ascorbate Peroxidase (APX)

Activity of APX enzyme was assayed as described by Sarkar et al. (2014). The reaction mixture was prepared by mixing potassium phosphate buffer (50 mM, pH 7.0), H_2_O_2_ (0.1 mM), and ascorbate (0.5 mM). The crude extract was added to the mixture to initiate the reaction and H_2_O_2_ -dependent oxidation of ascorbate was measured at 290 nm.

#### Catalase (CAT)

Catalase activity was determined by following the method of Sarkar et al. (2014). Briefly, the crude extract was mixed with potassium phosphate buffer (50mM, pH 7.5) and H_2_O_2_ (0.1 mM). The absorbance was measured at 240 nm. CAT activity was calculated on the basis of H_2_O_2_ utilization (extinction coefficient = 43.6 M^-1^cm^-1^) (Aebi, 1984).

#### Peroxidase (POD)

Peroxidase activity assay was conducted as described by Singh and Jha (2016). Briefly, the phosphate buffer (0.1 M, pH 7.0), pyrogallol (0.1 mM), and H_2_O_2_ (5 mM) were mixed with 100 μL of crude extract. The mixture was incubated at 25°C for 5 min. A 1.0 ml of 2.5 N H_2_SO_4_ was used to stop the reaction. The absorbance was read at 420 nm.

### Pathogenicity of *M. phaseolina* Strains under Field (Field Experiment)

Pathogenicity test of three fungal strains was conducted on the sunflower hybrids hysun 33 and SMH 0917 at the experimental fields of National Agriculture Research Center (NARC), Islamabad, Pakistan. The experiment was laid out in randomized completely block design (RCBD). The optimum temperature for sunflower was 70 to 78°F. Plants were sown with row to row distance of 75 cm and seeds were embedded in the ridges 1 to 2 cm with plant to plant distance of 12 cm. Each fungal strain was considered as single treatment with three replications and eight to ten plants per replication. The fungus was inoculated by tooth pick method (Simonetti et al., 2015). Briefly, the tooth picks (bamboo) were boiled for 30 min in a glass beaker and then dried on sterilized blotting paper. The dried tooth picks were transferred to three glass jars containing potato dextrose broth. Tooth picks were placed in jars vertically in such a way that half of the parts were dipped in broth. The pure culture of three pathogenic strains of *M. phaseolina* were poured into three jars with the help of cork borer and incubated at 28 ± 2°C for 14 days. After 14 days, tooth picks were ready to use.

At the flowering stage of sunflower plants, a hole was made in stem with a needle one foot above the soil surface. The tooth picks inoculated with respective fungus were incorporated into the holes. The inoculated plants were tagged with ribbons of different colors.

At physiological maturity, the disease was assessed by cutting the plant stem into two parts and measuring the symptoms up and down of the inoculated tooth picks. The infected part of the stem where microsclerotia developed was measured and scored on 0–6 scale, i.e., 1- up to 5 cm; 2- up to 10 cm; 3- up to 20 cm; 4- up to 30 cm; 5- up to 40 cm; 6- over 50 cm or completely wilted plant.

### Molecular Identification

The fungal strains were identified by 18S rRNA gene sequencing. The genomic DNA of the fungal pathogens was extracted by cetyltrimethyl ammonium bromide (CTAB) method (Brandfass and Karlovsky, 2008). Part of 18S rRNA region was amplified by using universal primers nu-SSU-0817-5′5′TTAGCATGGAATAATRRAATAGGA3′ and nu-SSU-1536-3′5′ATTGCAATGCYCTATCCCCA3′ (Borneman and Hartin, 2000). PCR reaction mixture consisted of 5 ng genomic DNA, 1.5 mM MgCl_2_, 10 μM of each primer, 1XTaq buffer (Fermentas) and 180–200 μM of each dNTPs. The reaction mixture was amplified in thermo cycler (Eppendorf) with the following amplifying conditions; Initial denaturation at 95°C for 5 min followed by 30 cycles of denaturation at 95°C for 45 s, annealing at 49°C for 45 s, extension at 72°C for 60 s and final extension at 72°C for 10 min. The amplified PCR product was analyzed on 1.2% agarose gel, purified by PCR purification kit (Fermentas) and sequenced by Macrogen Inc., Korea. Sequences were annotated and analyzed at BLAST to search the closest homolog.

### Random Amplified Polymorphism DNA (RAPD)

Random Amplified Polymorphic DNA analysis was used to detect the variations among the isolates of *M. phaseolina*. A total of six random primers of OPA series were used in this study (Prabhu et al., 2012). The sequences of primers used in the study are shown in the Supplementary Table S1.

### Phylogenetic Analysis

Full length Sequences of 18S rRNA were retreived from silva database. The sequences were aligned in Mega 6 with MUSCLE and clustered by using UPGMB method (Edgar, 2004). All sequences were trimed mannualy to remove a fair comparison with our own strains and realligned. The tree was constructed by neighbor joining to infer the evolutionary history (Saitou and Nei, 1987). The phylogeny was tested by applying bootstrap method (Felsenstein, 1985). The evolutionary analyses were conducted in MEGA6 which draw the tree to scale, with branch lengths in the same units as those of the evolutionary distances used to infer the phylogenetic tree and compute the by using the Maximum Composite Likelihood method (Tamura et al., 2004, 2013).

### Statistical Analysis

The data was analyzed with analysis of variance (ANOVA) using the statistical software MSTAT-C. The mean values were separated on the basis of Fisher’s least significant difference (LSD) test.

## Results

### Virulence of *M. phaseolina* Strains on Chick Pea (*Cicer arietinum*)

**FIGURE 1:**
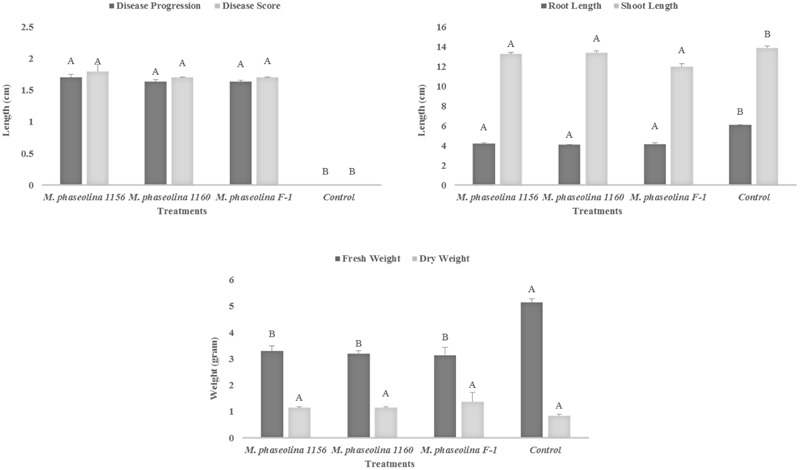
Growth retardation and disease progression in chickpea caused by *M. phaseolina.* Mean values followed by different letters are significantly different (*P* < 0.05).

All the *M. phaseolina* strains caused high virulence on chickpea (*Cicer arietinum*). A significant reduction in root length (RL) (44–49%), shoot length (SL) (5–16%), and fresh weight (55–63%) was observed in plants treated with pathogenic fungi as compared to that of untreated control (**Figure 1**). The dry weight of infected plants was found to be higher (26–38%) than that of healthy plants, i.e., (control). All the strains showed high virulence with a disease score 2 (**Figure 1**).

### Virulence of *M. phaseolina* Strains on Sunflower

**FIGURE 2:**
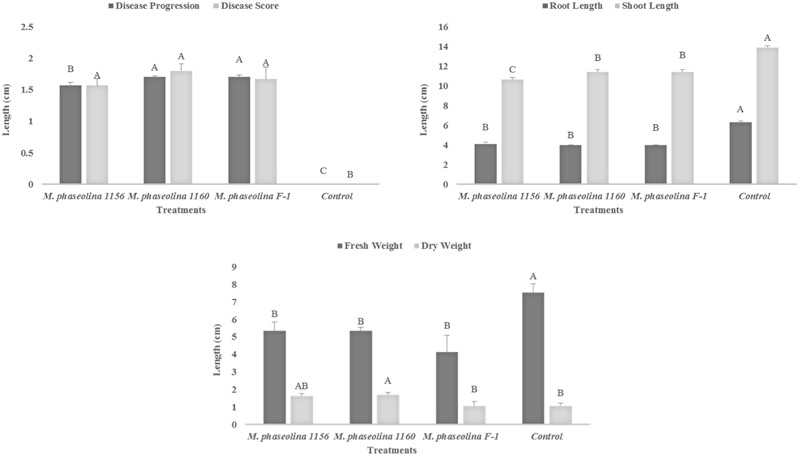
Growth retardation and disease progression in sunflower caused by *M. phaseolina.* Mean values followed by different letters are significantly different (*P* < 0.05).

The *M. phaseolina* strains caused disease score of 2 on sunflower plants in net house conditions (**Figure 2**) while a disease score of 5.8–6.0 under field conditions (**Table 1**). A significant reduction in RL (53–58%), SL (22–31%), and fresh weight (41–52%) was observed in sunflower plants inoculated with fungi as compared to that of control (**Figure 2**). The virulence of strains on sunflower hybrids under field conditions was highly significant (**Table 1**).

**Table 1:** Pathogenicity of *Macrophomina phaseolina* strains on sunflower under field conditions.

Fungal strains	Disease progression (DP)	Disease score (DS)
*M. phaseolina* strain PCMC/F1	81 A	6 A
*M. phaseolina* strain 1161	82 A	6 A
*M. phaseolina* strain 1156	81 A	6 A
No fungus (control)	0 B	0 B
**Hybrids**		
H1	82 A	6 A
H2	80 A	6 A

*Values are mean of three replications and separated by different letters in the same column are statistically different from each other.*

*H1 = Sunflower hybrid Hysun 33; Sunflower hybrid SMH-0917.*

### *In Planta* Pathogenicity Mechanism of *M. phaseolina*

Determinants of *in planta* pathogenicity and metabolic markers of oxidative stress viz H_2_O_2_ and other antioxidant enzymes were produced in both crops upon fungal infection.

#### Chick Pea (*Cicer arietinum*)

**FIGURE 3:**
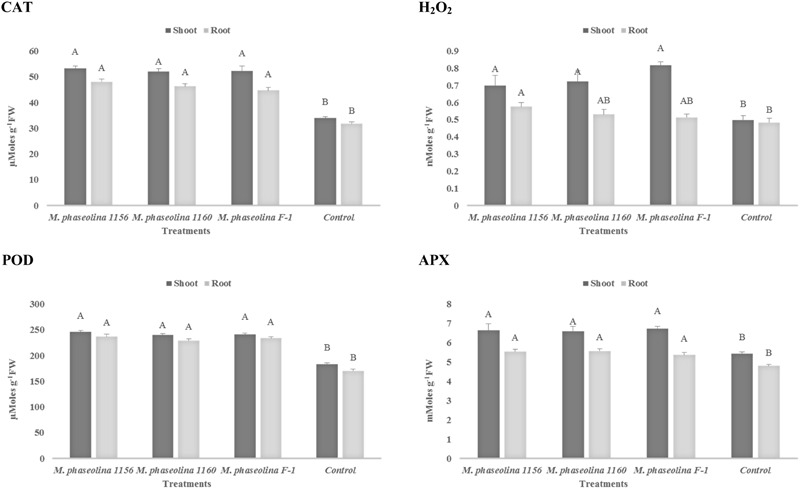
Activity of H_2_O_2_ and antioxidant enzymes in chickpea during *M. phaseolina* infection. Mean values followed by different letters are significantly different (*P* < 0.05). H_2_O_2_, hydrogen peroxide; CAT, catalase; POD, peroxidase; APX, ascorbate peroxidase.

A significant impact of *M. phaseolina* strains was observed on the H_2_O_2_ content and activity of ROS scavenging enzymes in chickpea. The H_2_O_2_ content was increased 1.4- to 1.6-fold in shoot of the plants treated with virulent strains of *M. phaseolina* over the untreated plants (**Figure 3**). Similarly the activities of ROS scavenging enzymes POD, APX, and CAT were increased 1.2- to 1.6-fold in the chickpea plants treated with *M. phaseolina* over untreated plants (control).

#### Sunflower (*Helianthus annuus* L)

**FIGURE 4:**
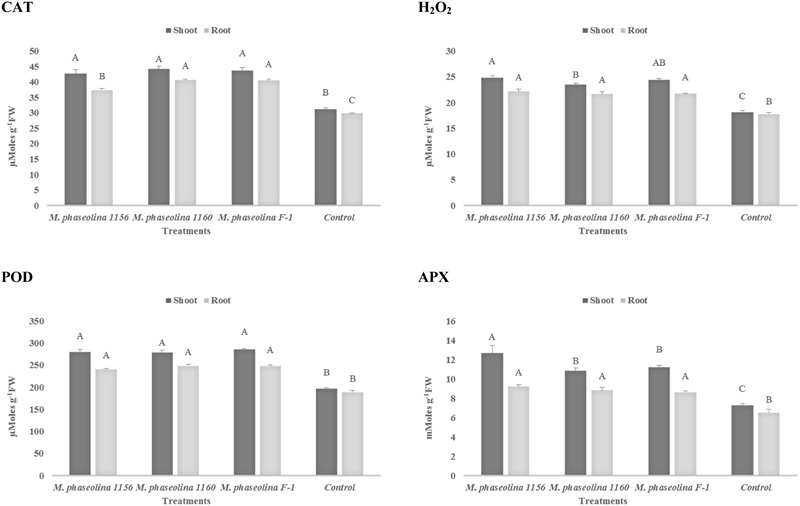
Activity of H_2_O_2_ and antioxidant enzymes in sunflower during *M. phaseolina* infection. Mean values followed by different letters are significantly different (*P* < 0.05). H_2_O_2_, hydrogen peroxide; CAT, catalase; POD, peroxidase; APX, ascorbate peroxidase.

**FIGURE 5:**
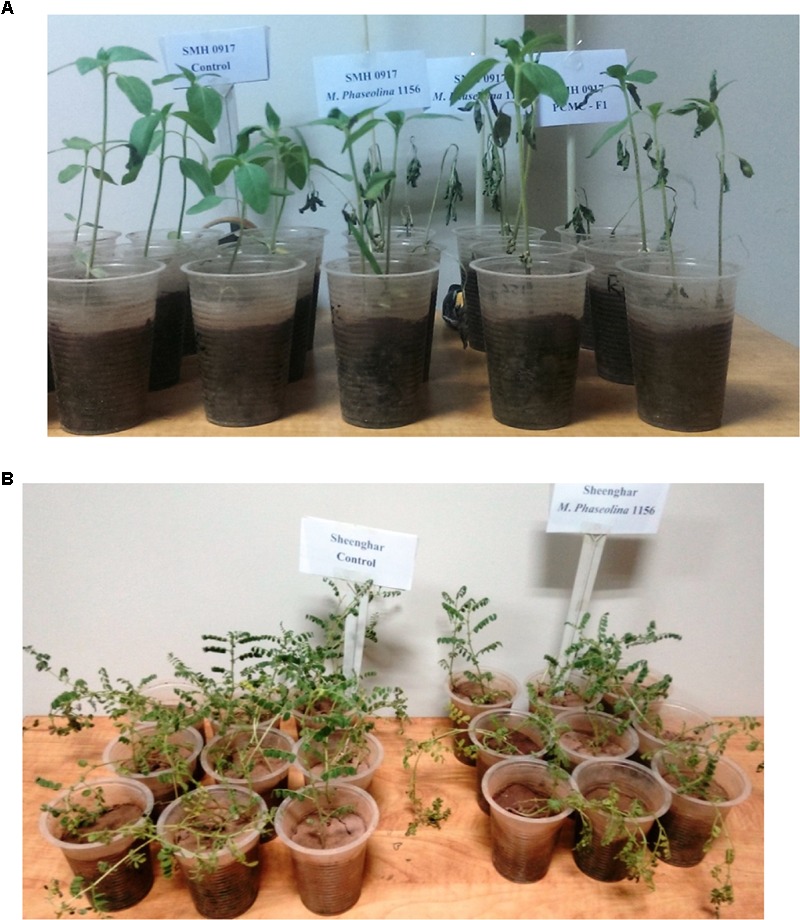
**(A)** Disease symptoms appeared on sunflower plants after *M. phaseolina* inoculation. **(B)** Disease symptoms appeared on chickpea plants after *M. phaseolina* inoculation.

In sunflower plants, *M. phaseolina* strains altered the concentration of H_2_O_2_ and the other enzymes in a similar trend as observed in chickpea. The H_2_O_2_ content was increased 1.2- to 1.3-fold in roots and 1.3- to 1.4-fold in shoots of the fungal treated plants over the untreated ones (control). The activities of ROS scavenging enzymes POD, APX, and CAT were also increased by 1.3- to 1.7-fold upon fungal infection (**Figure 4**). The phenotypic effect of *M. phaseolina* strains on sunflower and chick pea is shown in **Figures 5A,B**.

### Production of Hydrolytic Enzymes

**FIGURE 6:**
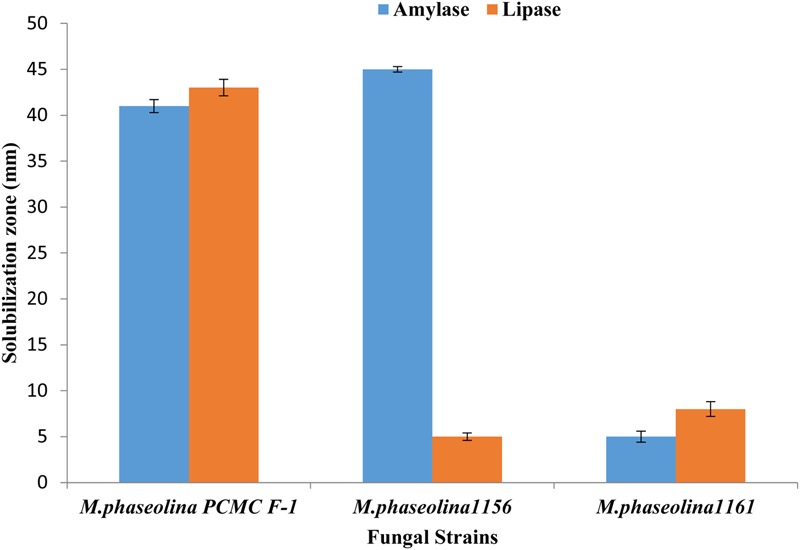
Production of hydrolytic enzymes by the *M. phaseolina* strains. Values are mean of three replications. Bars represent standard error.

*Macrophomina phaseolina* strains produced different hydrolytic enzymes. PCMC/F1 produced amylase and lipase with solubilization zone of 41mm and 43 mm, respectively (**Figure 6**). *M. phaseolina* strain 1156 also produced highest amylase with the solubilization zone of 45 mm but least lipase with the solubilization zone of 5 mm. The strain 1160 showed minimal production of amylase and lipase with a solubilization zone of 5 and 8 mm, respectively. Production of protease enzymes showed substrate specificity. The effect of substrates ‘starch’ and ‘gelatin’ was highly significant on the production of protease (**Table 2**). *M. phaseolina* strain 1160 showed maximum protease production with a solubilization zone of 35 mm followed by the strain PCMC/F1 and 1156 with a solubilization zone of 14 and 12 mm, respectively. Growth of fungus on respective substrate is shown in Supplementary Figure S3.

**Table 2:** Production of protease on different substrates by *M. phaseolina* strains.

Treatment	Solubilization zone (mm)
**Strain**	
*M. phaseolina* strain PCMC/F1	14.3 B
*M. phaseolina* strain 1160	35.3 A
*M. phaseolina* strain 1156	11.7 C
LSD _strains_	2.4
**Substrate**	
Gelatin	10.6 E
Starch	30.3 D
LSD _substrate_	2.0

*Values are mean of three replications and separated by different letters in the same column are statistically different from each other.*

### 18S rRNA Amplification and Accession No.

**FIGURE 7:**
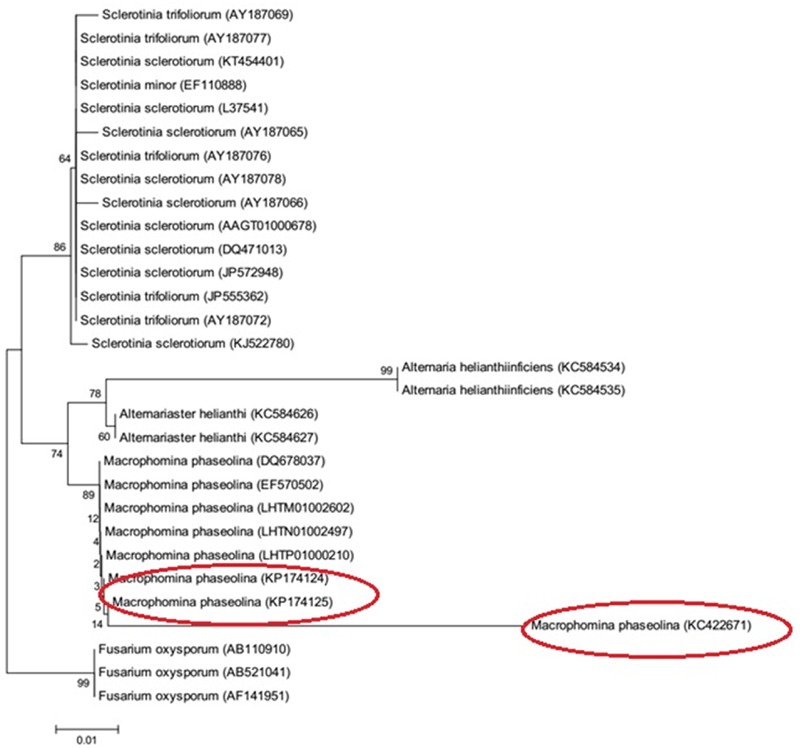
Phylogenetic tree of *M. phaseolina* strains based on the 18S rRNA gene.

A 762 bp of 18S rRNA gene was amplified as shown in Supplementary Figure S1. Sequences of 18S rRNA gene have been deposited in Gene Bank under the accession numbers KC422671, KP174124, and KP174125. Phylogenetic tree of *M. phaseolina* strains is shown in **Figure 7**. The strains revealed its relatedness with *M. phaseolina*. The strain PCMC was found to be different from other strains.

### RAPD Result (Dendrogram)

**FIGURE 8:**
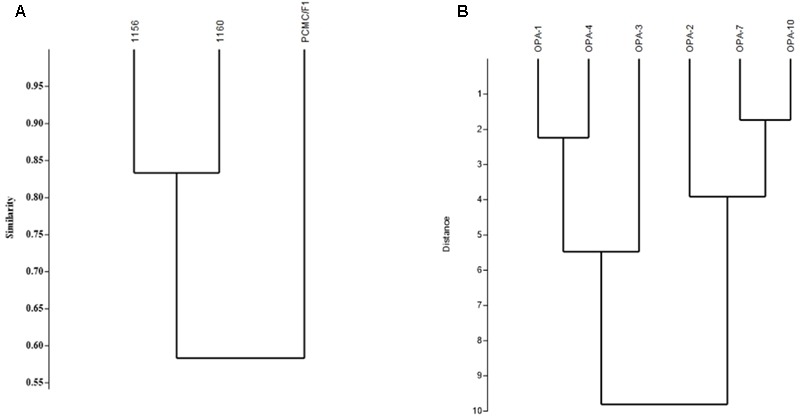
Genetic diversity of *M. phaseolina* strains based on the RAPD analysis. **(A)** Similarity index between *M. phaseolina* strains. **(B)** Similarity index between OPA primers.

Random Amplified Polymorphic DNA analysis showed genetic variations among the *M. phaseolina* strains. Out of the six primers used for amplification, OPA01 and OPA04 showed more than 80 percent polymorphism among isolates followed by OPA02 and OPA03 which showed 60 and 44% while OPA7 and OPA10 53 and 70% polymorphism. The Dendrogram constructed on the basis of band patterns using Jaccard’s coefficient in PAST software is shown in **Figure 8**. Different band patterns on agarose gel are shown in the Supplementary Figure S2.

## Discussion

*Macrophomina phaseolina* is one of the most devastating pathogens that infect more than 500 plant species throughout the world. It can grow swiftly in infected plants and afterward produces a large quantity of sclerotia that plugs the vessels, resulting in wilting of the plant. In present investigation, native strains of *M. phaseolina* isolated from sesame (*Sesamum indicum*), cotton (*Gossypium hirsutum*), and cowpea (*Vigna unguiculata*) showed highly virulence on chickpea and sunflower irrespective of their origin.

*Macrophomina phaseolina*, being a necrotrophic pathogen, kills the cells of host and reduces growth rate. In chick pea plant, we focused on root rot symptoms because pathogen enters through the roots leading to the appearance of symptoms firstly on the roots. While in sunflower, basal node is the entry point of this pathogen. The *M. phaseolina* strains reduced the growth of root and shoot of both crops without any significant difference among their virulence.

Host adaptability of *M. phaseolina* strains has been found to be dependent on many factors. The adaptation of soybean associated strains was highly impacted by crop rotation while other strains showed specificity with corn (Su et al., 2001; Almeida et al., 2008). These findings depict the diversity in different *M. phaseolina* strains. In the present era of intensive cultivation and irrational use of fertilizers/pesticides, may tend toward the emergence of new races of the strains.

*Macrophomina phaseolina* strains significantly enhanced the quantities of metabolites/enzymes causing oxidative stress in cell. As the necrotrophic pathogens prefer to feed on cell remain, they kill the host cells through creation of oxidative stress (Laluk and Mengiste, 2010). Thus, measurement of oxidative stress determinants could be ideal to estimate *in planta* pathogenicity mechanism of phytopathogens.

A higher quantity of H_2_O_2_ was observed during the *M. phaseolina* infection on both hosts. H_2_O_2_ is an important component of ROS and found to be associated with virulence of numerous pathogens (Yu et al., 2017).

In oxidative stress, ROS scavengers and antioxidant enzymes are highly activated to neutralize the negative effect. The fungal inoculation also enhanced the activity of ROS scavenging enzymes CAT, POD, and APX which advocated the presence of high oxidative stress during fungal infection. Similar findings on other hosts under different pathogens stress have been reported (Anthony et al., 2017).

In our study, a consistency between the phenotypic symptoms and biochemical changes was observed depicting that *M. phaseolina* cause virulence by creating strong oxidative stress in the host cells.

The strains also showed variable potential to secret hydrolytic enzymes viz pectinase, amylase, and protease. Hydrolytic enzymes are secreted by virulent strains of pathogens which they utilize to overcome the physical barriers by degrading various cell wall components of plants like pectin, lipid, cellulose, and proteins (Wheeler, 1975). In present study, all the *M. phaseolina* strains produced amylases. This is in contrast to the earlier report in which *Macrophomina* sp. was unable to produce amylase (Sohail et al., 2009). However, the strains produce variable amount of protease which were consistent to the earlier findings (Ahmad et al., 2006).

Production hydrolytic enzymes by the pathogenic fungi have been reported in determining their pathogenic potential (Ghannoum, 2000; Lawrence et al., 2000; López-Otín and Overall, 2002). These enzymes not only facilitate the fungal penetration by dissolving different plant structures like peptide bonds, phospholipids but also act as signaling molecules for the induction of HR (Sarkar et al., 2014). The proteolytic processing mediated by protease enzyme, cause change in certain protein function, and used as common mechanism for controlling the biological processes at cellular level (López-Otín and Overall, 2002).

Sequence of 18S rRNA of all the strains showed closest homology, i.e., 99% similarity with the reference sequences deposited in Gene Bank and confirmed their identification as *M. phaseolina.* The clustering of 18S rRNA sequences of test isolates with that of “Gene Bank reference sequences” further validated the identification results. A greater similarity in a 490–491 bp of 18S rRNA gene validated its conserved nature among the *M. phaseolina* strains. The Identification of fungi based on 18S rDNA is authenticated and well reported (Romanelli et al., 2014).

Phylogenetic analysis delineated the strains 1156 (KP174124) and 1160 (KP174125) were into same clades while strain PCMC F1 (KC422671) into different clade. These findings showed that *M. phaseolina* is not confined to either geographical location and/or a specific host. The distribution of *M. phaseolina* groups have been found to be independent of sampling location and host in numerous studies except a few reports in which *M. phaseolina* strains exhibited host specificity (Jana et al., 2003; Purkayastha et al., 2006; Bashasab and Kuruvinashetti, 2007; Rayatpanah et al., 2009; Baird et al., 2010; Saleh et al., 2010).

DNA markers such as RAPDs using polymerase chain reactions (PCR) have been widely used for detecting genetic diversity among the microorganisms (Sharma et al., 2013). In present study, RAPD analysis clearly indicated high polymorphism among the *M. phaseolina* strains depicting its effectiveness in evaluation of genetic diversity in *M. phaseolina*.

The genetic diversity within strains of *M. phaseolina* have been widely studied using the RAPD and rDNA sequencing (Jana et al., 2003; Purkayastha et al., 2006; Bashasab and Kuruvinashetti, 2007; Babu et al., 2010a; Saleh et al., 2010; Sundravadana et al., 2011; Mudalige et al., 2012).

These reports on the basis of RAPD markers and 18S rRNA sequence analysis clearly indicate genetic variation among the strains collected from different hosts and shore up the possibility of emergence of various pathotypes in *M. phaseolina* independent of host specificity. Variation in the pathogenicity may be associated with their ability to produce hydrolytic enzymes and genetic diversity. The broad host range enables *M. phaseolina* to survive in soil for a long time and cause great damage to all the crops. Therefore, there is need to design the new control strategies for the management of emerging genetically diverse pathotypes of *M. phaseolina.*

## Author Contributions

AK performed the RAPD PCR analysis and involved in write up. FS analyzed the RAPD data and was involved in writing the manuscript, KM conducted pathogenicity test on sunflower plants. ZH helped in setting up the PCR experiments. MK monitored the field experiments. FH edited the manuscript and co supervised the experiments. MH designed the study, edited and revised the manuscript and supervised the experiments conducted at his laboratory.

## Conflict of Interest Statement

The authors declare that the research was conducted in the absence of any commercial or financial relationships that could be construed as a potential conflict of interest.
