# Supplementary figures and images for: Molecular Identification and Genetic Characterization of Macrophomina phaseolina Strains Causing Pathogenicity on Sunflower and Chickpea
Source: Front Microbiol. 2017 Jul 19;8:1309. doi: 10.3389/fmicb.2017.01309 (PMC5515817; doi:10.3389/fmicb.2017.01309)

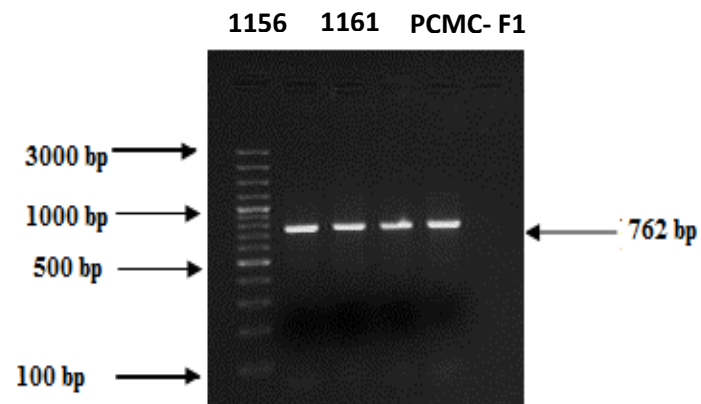

**Figure S1: Amplification of 18S rRNA from the *M. phaseolina* strains.**

Supplement: Supplementary file 1 [file Image_1.pdf]
