# Supplementary material for: Molecular Identification and Genetic Characterization of Macrophomina phaseolina Strains Causing Pathogenicity on Sunflower and Chickpea
Source: Front Microbiol. 2017 Jul 19;8:1309. doi: 10.3389/fmicb.2017.01309 (PMC5515817; doi:10.3389/fmicb.2017.01309)
Supplement: Supplementary file 2 [file Image_2.pdf]

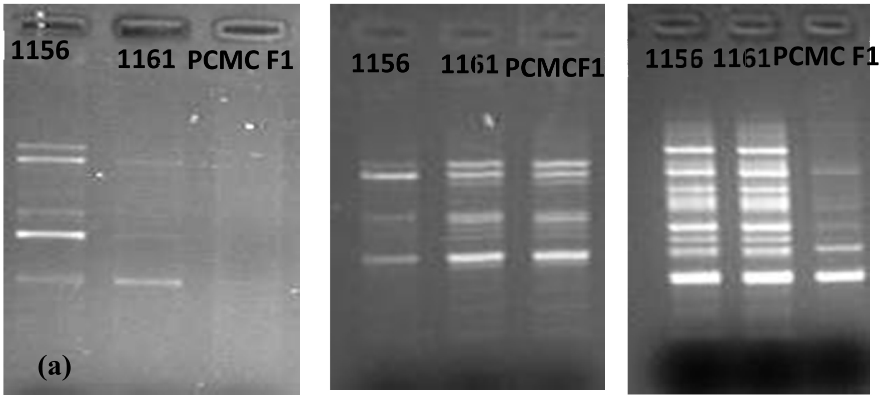

(a)OPA-1 (b) OPA-2 (c) OPA-3

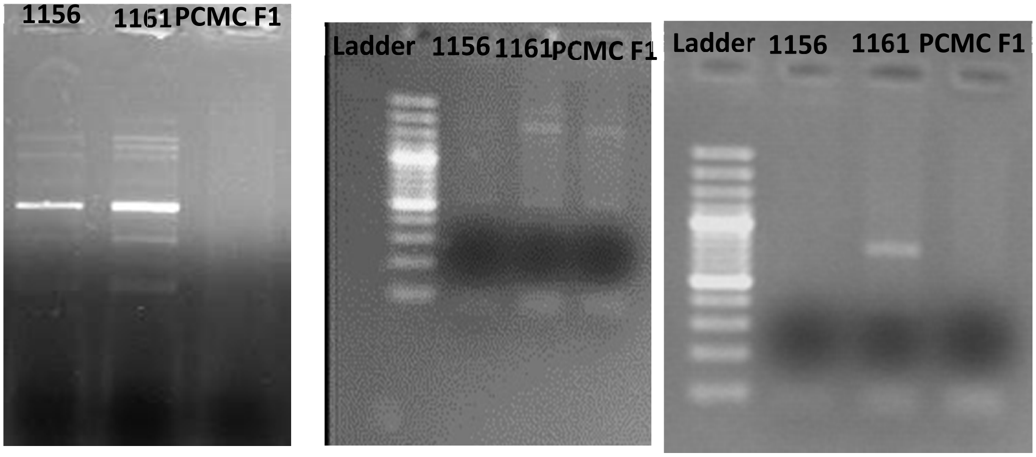

(d) OPA-4 (e) OPA-7 (f) OPA-10

Figure S.2. PCR Profile of *M. phaseolinia* strains amplified with RAPD primers

a-f: PCR amplification with different primers
