# Supplementary material for: Molecular Identification and Genetic Characterization of Macrophomina phaseolina Strains Causing Pathogenicity on Sunflower and Chickpea
Source: Front Microbiol. 2017 Jul 19;8:1309. doi: 10.3389/fmicb.2017.01309 (PMC5515817; doi:10.3389/fmicb.2017.01309)
Supplement: Supplementary file 3 [file Image_3.pdf]

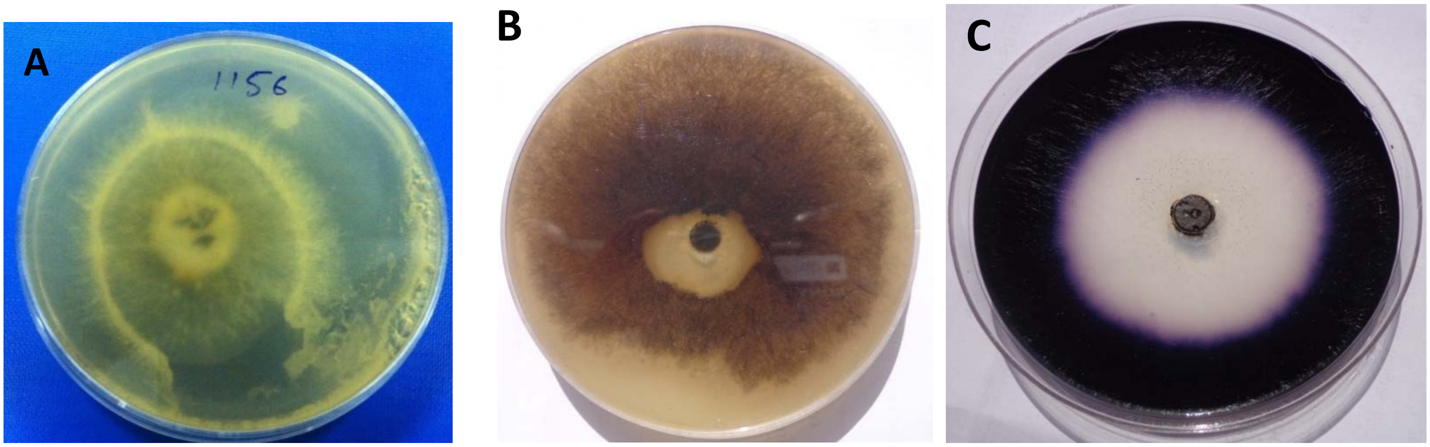

Figure S3. Production of hydrolytic enzymes by *M. phaseolina* strains.

- **(A) Protease production on skim milk agar by *M. phaseolina* strain 1160. The zone was detected by observing transparent area appeared after degradation of casein.**
- **(B) Lipase production on rhodamine B agar by *M. phaseolina* strain PCMC-F1. The production zone was observed on the basis of yellow-orange colour appeared under UV light.**
- **(C) Amylase production on soluble starch by *M. phaseolina* strain 1156. The production zone was detected by spreading 1% lugol solution on the plate.**
