# Supplementary material for: Molecular Identification and Genetic Characterization of Macrophomina phaseolina Strains Causing Pathogenicity on Sunflower and Chickpea
Source: Front Microbiol. 2017 Jul 19;8:1309. doi: 10.3389/fmicb.2017.01309 (PMC5515817; doi:10.3389/fmicb.2017.01309)
Supplement: Supplementary file 4 [file Table_1.pdf]

Table S1. Sequences of the primers

Primers	Sequence
OPA-01	CAGGCCCTTC
OPA-02	TGCCGAGCTG
OPA-03	AGTCAGCCAC
OPA-04	AATCGGGCTG
OPA-07	GAAACGGGTG
OPA-10	GTGATCGCAG
